# Bilateral Nipple Enlargement as a Secondary Effect of Anabolic Drugs: A Histopathological Mimicker of Smooth Muscle Hamartoma

**DOI:** 10.3390/dermatopathology8020016

**Published:** 2021-04-15

**Authors:** Mar Llamas-Velasco, Maria Francesca Bianciardi Valassina, Enrique Ovejero-Merino, Guido Massi, Thomas Mentzel

**Affiliations:** 1 Department of Dermatology, Hospital Universitario de La Princesa, 28006 Madrid, Spain; 2 Voth Laboratorio Diagnóstico, 28006 Madrid, Spain; enrique.ovejero@gmail.com; 3 Plastic Surgeon, Circ. Aurelia 19, 00136 Rome, Italy; francescabianciardi@libero.it; 4 Thoracic Surgery, Hospital Universitario de la Princesa, 28006 Madrid, Spain; 5 General Surgery, Hospital Universitario Príncipe de Asturias, 28006 Madrid, Spain; 6 Dermodiagnostica, Via Festo Avieno, 60, 00100 Rome, Italy; patologiacutanea@gmail.com; 7 Dermatopathologie Friedrichshafen, 88048 Friedrichshafen, Germany; mentzel@dermpath.de

**Keywords:** smooth muscle hamartoma, anabolic agents, nipples, hamartoma, hyperplasia

## Abstract

Smooth muscle hamartoma are usually solitary and congenital, may affect the genital area and nipples. Histopathologically, they are characterized by the presence of mature smooth muscle bundles. We present a 40 year-old male with bilateral nipple enlargement excised with clinical suspicion of bilateral leiomyoma. Skin biopsy shows mature, irregularly arranged smooth muscle bundles and lactiferous ducts between them. Immunohistochemistry is positive for smooth muscle actin, desmin and fumarase, but negative for estrogen and progestogen receptors. The presence of lactiferous ducts excludes bilateral leiomyomas. Even when, histopathologically, this can be interpreted as the nipple-type of muscular hamartoma of the breast, clinical history favors an anabolic drug-induced lesion. Bodybuilders present gynecomastia and nipple enlargement as frequent problems, but we have not found any histopathological description of these nipple lesions. We consider that dermatologists should be aware of the presence of them and dermatopathologists should know their histopathological features to avoid misdiagnosis as neoplasms.

## 1. Introduction

Smooth muscle hamartoma is usually a solitary and congenital lesion, mostly involving the back and lower limbs, although the mammary region may be affected [1]. Clinically, the commoner presentation is as a single skin-colored or hyperpigmented hairy lesion, even when it may present a gamut of appearances as morphea-like lesions, follicular spotted ones, vascular-like ones [2] or in a generalized pattern termed “Michelin tire baby” [3]. Histopathologically, it shows a disorganized proliferation of mature well-demarcated bundles of smooth muscle without spatial relation with hair follicles [4].

We present a bilateral enlargement of both nipples, histopathologically mimicking a smooth muscle hamartoma, but containing additionally lactiferous ducts.

## 2. Case Report

A 40-year-old male presented with progressive bilateral enlargement of both nipples excised due to cosmetic concerns. No medical relevant history, but intake of anabolic steroids to improve his physical performance.

**Figure 1:**
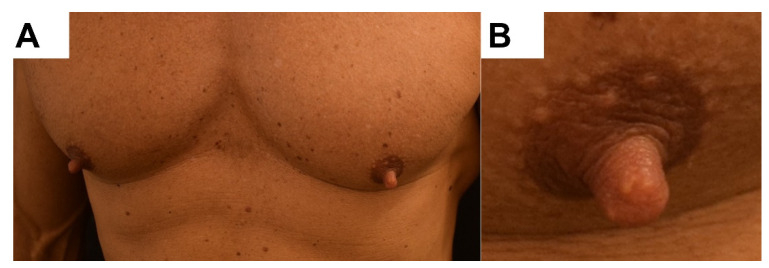
Clinical picture. (**A**) Bilateral and quite symmetrical enlargement of both nipples. (**B**) Elastic consistency of a nipple with a length of 11 mm.

Physical examination showed symmetrical, cylindrical, well-defined and slightly indurated nipples of 10 mm diameter and 11 mm height, which were excised with the clinical diagnosis of bilateral leiomyomas (Figure 1).

**Figure 2:**
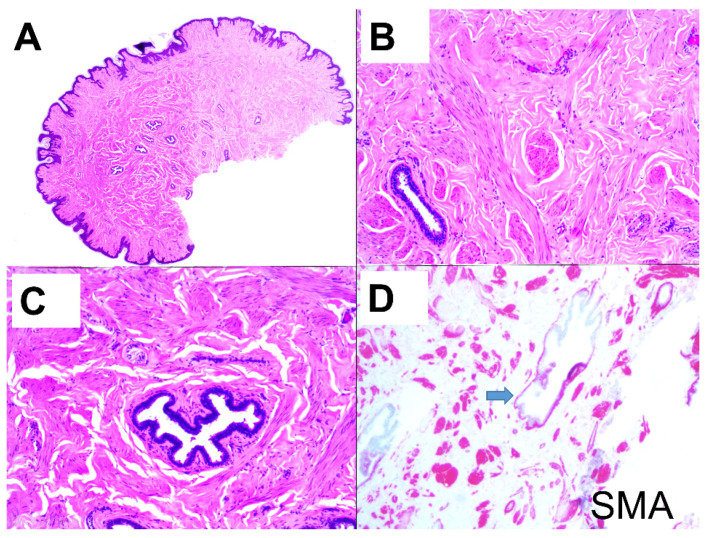
Histopathological picture. (**A**) The panoramic picture shows irregularly arranged smooth muscle bundles, a slightly papillomatous epidermis, and some dilated ducts in the deeper areas. (**B**) Close view of the irregularly arranged smooth muscle bundles. (**C**) Closer view of a lactiferous duct surrounded by myoepithelial layer. (**D**) HEx4 picture of the lesion stained with smooth muscle actin (SMA) shows the smooth muscle’s global distribution. A blue arrow shows myoepithelial cells.

Skin biopsy shows a slightly hyperplastic epidermis and haphazardly arranged fascicles of smooth muscle within the reticular dermis (Figure 2A). No cytological atypia or necrosis is present (Figure 2B). Intermingled with these smooth muscle fascicles appear, in different orientations, numerous glandular structures composed of an inner cylindric layer, showing prominent apocrine features and a flattened outer layer of myoepithelial cells (Figure 2C).

Immunohistochemistry shows that smooth muscle (SMA) is positive with actin (Figure 2D) and desmin, and there is a preserved expression of fumarase. P63 focally stains myoepithelial cells that are also actin positive. Estrogen and progesterone receptors are negative.

With the combined clinical and histopathological appearance, we diagnose bilateral hamartomatous lesion of the nipple, probably induced by anabolic drugs.

## 3. Discussion

The main differential diagnosis in our case is bilateral leiomyoma of the nipple, a rarely reported problem [5,6]. Clinically, both entities appear as a progressive enlargement of the nipple. Histopathologically, in leiomyomas, interlacing bundles of smooth muscle fibers without necrosis, nuclear atypia or mitosis and with no or minimal fibrous tissue and a complete absence of glandular elements are observed [7]. Thus, this diagnosis can be easily ruled out in our case as our patient presented numerous lactiferous ducts.

On the other hand, smooth muscle hamartoma can be associated with vascular lesions [8,9,10] or melanocytic lesions [11,12,13] and even with glandular structures [14], although we have not found any previously reported case with lactiferous glands.

Therefore, our case is noteworthy, as histopathological findings fit better within the concept of a complex hamartomatous lesion of the nipple as hamartomas are defined as benign tumoral nodules composed of an overgrowth of mature cells and tissues normally found in the affected area, and we observed mostly smooth muscle fascicles and lactiferous glands. Moreover, our patient’s nipple lesions can be considered as the nipple-type analogous to the widely known muscular hamartoma of the breast [15].

From a clinicopathological point of view, our patient’s lesions appeared chronologically related to anabolic drug intake. This problem has been described mostly in plastic surgery articles, seems to be commoner in Asian females, sometimes related with hormonal alterations and, as this is considered mainly an esthetic problem. Thus, as insurance companies do not usually cover the pathological study in these case, this fact could explain why we have not found any histopathological report similar to ours [16,17].

There are previous articles on the capacity of hormones to induce smooth muscle growth in nipple leiomyomas [5] with immunohistochemical differences regarding estrogen and progesterone receptor staining. Moreover, anabolic androgenic steroids abuse has a global lifetime prevalence of up to 6.4% in males and, due to their negative feedback in the hypothalamic-pituitary-gonadal axis, can cause gynecomastia through a decrease in LH (Luteinizing hormone), FSH (Follicle-stimulating hormone) and testosterone serum levels [18,19] and, through this pathway, an abnormal stimulation of nipple tissue seems to be plausible. Nipple tissue may present dermal stem cells with the capacity of differentiating into divergent cell lineages, including smooth muscle and glandular structures under the influence of hormonal stimulus. Due to these facts, in our nipple hamartomatous lesions, a hormonally driven etiology seems to be the most probable etiology.

## 4. Conclusions

In conclusion, dermatopathologists, as other physicians, should also increase their awareness of problems associated with doping drugs like anabolic steroids and, even when these lesions are frequently excised due to cosmetic concerns, the histopathological study should be done to increase the knowledge on these lesions.
